# Inflammation Is a Key Pathophysiological Feature of Metabolic Syndrome

**DOI:** 10.1155/2013/135984

**Published:** 2013-04-18

**Authors:** Fabrizio Montecucco, François Mach, Aldo Pende

**Affiliations:** ^1^Division of Cardiology, Department of Internal Medicine, Foundation for Medical Researches, University of Geneva, 64 Avenue de la Roseraie, 1211 Geneva, Switzerland; ^2^Laboratory of Phagocyte Physiopathology and Inflammation, Department of Internal Medicine, First Medical Clinic, University of Genoa, 6 Viale Benedetto XV, 16100 Genoa, Italy

Metabolic syndrome is a disease clustering different cardiovascular risk disorders, including central obesity, insulin resistance, glucose intolerance, dyslipidemia, and hypertension. In the last decade, different definitions have been produced to better assess the multiplicative cardiovascular risk of metabolic syndrome and potentially improve patient treatment in both primary and secondary prevention. In particular, insulin resistance progressively lost its relevance on metabolic syndrome patients, being considered as a concomitant rather than a diagnostic condition. On the other hand, local organ remodeling and structural alterations (including adipose tissue, heart, liver, and pancreatic *β*-cells) in metabolic syndrome have been targeted by pathophysiological studies. Given the worldwide pandemic dimensions of metabolic syndrome, a strong scientific effort has been made on the identification of the causal mechanisms and molecules underlying this disease. In addition, both basic research and clinical studies investigating prognostic circulating biomarkers of metabolic syndrome have shown some soluble mediators (such as inflammatory cytokines, adipocytokines, and coagulation factors) as crucial players in the development of metabolic syndrome. More recently, the alterations in free fatty acid metabolism have also indicated these molecules as critical mediators of inflammation in metabolic syndrome. This special issue was focused on these and other soluble mediators as promising candidates to better assess the cardiovascular risk in metabolic syndrome. In particular, the pathophysiological activities of adipocytokines, cytokines, and chemokines as well as the controversies on the role of insulin resistance have been discussed. F. Renna et al. investigated the role of the enzyme cyclooxygenase-2 (COX-2) in an experimental rat model of spontaneous hypertension and metabolic syndrome. Using the COX-2 specific antagonist lumiracoxib, the authors showed that the treatment with this drug was able to reverse vascular remodeling and inflammation, confirming the potential role for COX-2 in the pathophysiology of atherogenesis. H. Qu et al. investigated the potential relationship between a novel systemic biomarker (i.e., plasma progranulin [PGRN]) as well as interleukin- (IL-) 6 with insulin resistance in Chinese patients with normal glucose tolerance (*n* = 88) and type 2 diabetes (*n* = 80). Results showed that systemic PGRN concentrations were significantly increased in the diabetic patients as compared to normal glucose control group. Importantly, PGRN levels were positively related with patient weight, central obesity, inflammation, and insulin resistance (assessed by HOMA-IR). C. L. Reading et al. focused on the potential anti-inflammatory benefits of the insulin sensitizer, HE3286. Importantly, this drug was able to decrease insulin resistance in metformin-treated subjects, indicating a therapeutic potential to restore metabolic homeostasis in type 2 diabetes. O. Bădulescu et al. investigated the potential association between diabetes mellitus and other cardiovascular risk factors (such as dyslipidemia) in three different groups of patients: diabetic without ischemic-cardiopathy-related disorders, diabetic with clinical ischemic cardiopathy, and nondiabetic with ischemic-cardiopathy-related disorders, respectively. The results showed that diabetic patients have an increased thrombotic risk as well as highest levels of interleukin-1-beta and lipids. Therefore, this study shows that the diagnostic conditions of metabolic syndrome might differently affect the cardiovascular risk in human beings. F. Rodriguez et al. investigated the role of the adipocytokine resistin on different aspects (such as inflammation, food intake, and gonadal function) both *in vitro* in rat adenopituitary cells and *in vivo *in fed and fasting rats. The authors showed that exogenous administration of resistin increased *β*-oxidation and inhibited metabolic enzymes involved on lipid synthesis. This study indicated that resistin might have a regulatory and homeostatic role on lipid metabolism within the pituitary gland.

F. Wasinski et al. investigated the potential benefits of physical exercise and caloric restriction *in vivo* on inflammatory cells, resident within adipose tissue, in obese mice. Both exercise and caloric restriction improved body mass, number of resident immune cells in the adipose tissue, and serum levels of inflammatory molecules in obese animals. However, selective mediators were modified by these therapeutic approaches, indicating potential different antiinflammatory pathways, suggesting that the combination of both strategies might be very promising to reduce inflammation in obesity. Potential controversies on the role of obesity on the cardiovascular risk have been particularly debated. B. K. Cole et al. investigated the inflammatory role of 12/15-lipoxygenase activity in white adipose tissue from obese mice with fat-specific deletion of this enzyme. Knockout mice showed improvements in fasting glucose levels and metabolism when compared to controls. In addition, a reduced inflammation and macrophage infiltration characterized adipose tissue of knockout mice. These results suggest that specific deletion of 12/15-lipoxygenase in adipose tissue can protect from the deleterious effects of inflammation. Two articles have also been included about a condition accelerating atherosclerosis. A review article by I. Ferraz-Amaro et al. focused on potential common mediators between metabolic syndrome and chronic inflammatory diseases, such as rheumatoid arthritis (RA). The authors provided a complete overview on the potential role of adipocytokines that could influence atherogenesis in both diseases. Biological therapies, including anti-TNF-*α* drugs, have been indicated to potentially improve atheroprogression and insulin resistance in RA patients. Finally, P. H. Dessein et al. performed a clinical study on 277 black African subjects (of whom 119 had RA) investigating the potential association between adipocytokine serum levels, RA, and lipid profile. Only in RA subjects, adiponectin concentrations were associated with a favorable lipid profile and blood pressure. No associations were observed for leptin. This clinical study partially confirmed a potential pathophysiological relevance for adiponectin in chronic inflammatory diseases. The present special issue on mediators of inflammation represents an update on pathophysiological mediators that can be targeted by novel and more selective treatments in metabolic syndrome and concomitant chronic inflammation. We hope that the reader will find some novel input for future researches.

*Fabrizio Montecucco* *Fabrizio Montecucco* *François Mach* *François Mach* *Aldo Pende* *Aldo Pende*

